# Iron chelation as a therapeutic target in vanadium neurotoxicity and Parkinson's disease: role of medicinal plants

**DOI:** 10.3389/fneur.2025.1667943

**Published:** 2025-10-24

**Authors:** Francis Olaolorun, Melanie-Jayne R. Howes, Taiwo Elufioye, Oluwatoyin A. Odeku, James Olopade, Paul Chazot

**Affiliations:** ^1^Neuroscience Unit, Department of Veterinary Anatomy, University of Ibadan, Ibadan, Nigeria; ^2^Department of Biosciences, Durham University, Durham, United Kingdom; ^3^Royal Botanic Gardens Kew, Richmond, United Kingdom; ^4^Department of Pharmacognosy, Faculty of Pharmacy, University of Ibadan, Ibadan, Nigeria; ^5^Department of Pharmaceutics and Industrial Pharmacy, University of Ibadan, Ibadan, Nigeria

**Keywords:** *Spondias*, iron homeostasis, PINK-1, *Drosophila*, oxidative stress, Parkinson's disease

## Abstract

Bioprospecting plant natural products has yielded significant success in the development of symptomatic treatment of neurodegenerative diseases, including the two most common, Alzheimer's and Parkinson's diseases (PD). Dysregulation of iron has been strongly implicated in the pathophysiology of these serious intractable diseases. A series of Nigerian endemic plants' methanolic extracts were explored using a Ferrozine binding iron chelation assay. This identified *Spondias purpurea* L. (SP) leaves as a potential therapeutic candidate and this was determined by evaluation of oxidative stress in 6-hydroxydopamine (6-OHDA)-exposed monoamine cell culture and *Drosophila* models of PD and vanadium neurotoxicity. SP treatment protected CAD cells against 6-OHDA toxicity and improved survival in PINK-1 mutant flies, though it had little effect on motor deficits. Furthermore, SP treatment reduced the vanadium-induced reactive oxygen species, and notably, staggered SP treatment significantly extended lifespan in vanadium-treated flies. Overall, *Spondias purpurea* L. leaf methanolic extract exhibited iron-chelating, antioxidant, neuroprotective, and life-extending properties, relevant to Parkinson's disease and vanadium-induced toxicity.

## 1 Introduction

Globally, there is an ongoing demographic shift toward increased life expectancy. Compared to the 1950s when old people (people > 65 years of age) accounted for about 5% of the world population, it is projected that by 2050 the figure will be around 16.7% (1). While advances in technology and medicine are helping to increase human lifespan, there has not been a corresponding improvement in “healthspan”— the number of years spent in good health (2). As a result, age associated diseases such as cancer, diabetes, cardiovascular diseases, dementia, etc. are gaining prominence worldwide. Age is a major risk factor in the development of neurodegenerative diseases (NDs) such as Alzheimer's disease (AD) and Parkinson's disease (PD) (3), with about 70% of people living with dementia being above 75 years of age (4).

Beyond age, exposure to environmental pollutants, such as heavy metals, represents another significant risk factor in the development of PD (5, 6). Vanadium is one such heavy metal of particular concern (7)—a metal widely distributed in the earth crust and a major contaminant in fossil fuels. Vanadium exposure can occur in occupational settings within vanadium-allied industries or through environmental pollution arising from gas flaring, crude oil spills or other crude oil exploration related incidents (8, 9). Studies in humans and experimental animal models have demonstrated vanadium's neurotoxic properties as reviewed by (10), including its ability to cause earlier onset of aging-associated neuropathologies (11). Via its ability to inhibit phosphatases, vanadium is able to induce hyperphosphorylation of tau at serine394, and this has been proposed as a potential mechanistic link between vanadium exposure and tauopathies (12).

Parkinson's disease is the fastest growing, and second most common ND worldwide (13). The early clinical presentation involves motor impairments (bradykinesia, muscle rigidity, and resting tremor) and cognitive decline at latter stages of the disease (14). The pathological hallmark of PD is the progressive loss of dopaminergic neurons in the substantia nigra pars compacta (SNpc), accompanied by abnormal intraneuronal accumulation of Lewy bodies in the SNpc and other brain regions (15). Currently, there is no cure for PD—only symptomatic treatments are available, and these do not ultimately slow disease progression (16, 17). Despite the association of most NDs with accumulation of misfolded protein aggregates, therapeutic strategies targeting clearance of such aggregates are yet to give satisfactory results. This has prompted continued investigation into alternative therapeutic targets.

Iron accumulates regionally in the aging brain and has been specifically shown to accumulate in brain regions associated with PD (18, 19). This iron accumulation contributes to oxidative stress and cellular damage (20), with iron levels in the SN correlating with disease severity in PD patients (21, 22). Iron can induce the conformational transformation of α-synuclein—the principal protein implicated in PD—from its α-helix form to the β-sheets typically found in Lewy bodies (23–25). α-synuclein mRNA has a putative iron regulatory element (IRE) at its 5′ untranslated region, suggesting that intracellular iron levels may regulate its translation, although this relationship is yet to be conclusively demonstrated (26). Furthermore, several proteins involved in iron metabolism are reportedly dysregulated in PD. Ferritin levels in the SN of PD patients are significantly lower than in age-matched controls (27), while divalent metal transporter 1 (DMT1) expression is increased in the SN of PD patients (28). These changes may contribute to the elevated iron content in the brains of PD patients and could contribute to the disease progression.

Iron has also been implicated in mitochondrial dysfunction— an important pathophysiological feature of PD. The relationship between iron and mitochondria dysfunction is bidirectional: excess iron can cause mitochondrial fragmentation (29), while mitochondrial dysfunction can promote iron accumulation via increased iron regulatory protein 1 (IRP1) activity and elevated expression of DMT1 and transferrin receptor 1 (TfR1) (30). The importance of iron in PD pathogenesis is increasingly being recognized, as evidenced by recent successes of iron chelating therapies in PD experimental models and clinical trials (31). Iron plays a central role in multiple pathological processes associated with PD, including α-synuclein aggregation, oxidative stress, and mitochondrial dysfunction (26). Additionally, iron modulates vanadium toxicity (10, 32, 33). Given these interconnected mechanisms, we investigated three plants used medicinally in Nigeria (*Carpolobia lutea* G. Don*, Phyllanthus muellerianus* (Kuntze) Exell and *Spondias purpurea* L.), for their iron chelating properties and therapeutic potential in cell culture and *Drosophila* models of PD and vanadium neurotoxicity. These plants were selected based on their documented antioxidant, anticholinesterase and neuroprotective properties (34–36).

## 2 Results

### 2.1 Iron chelation

**Figure 1:**
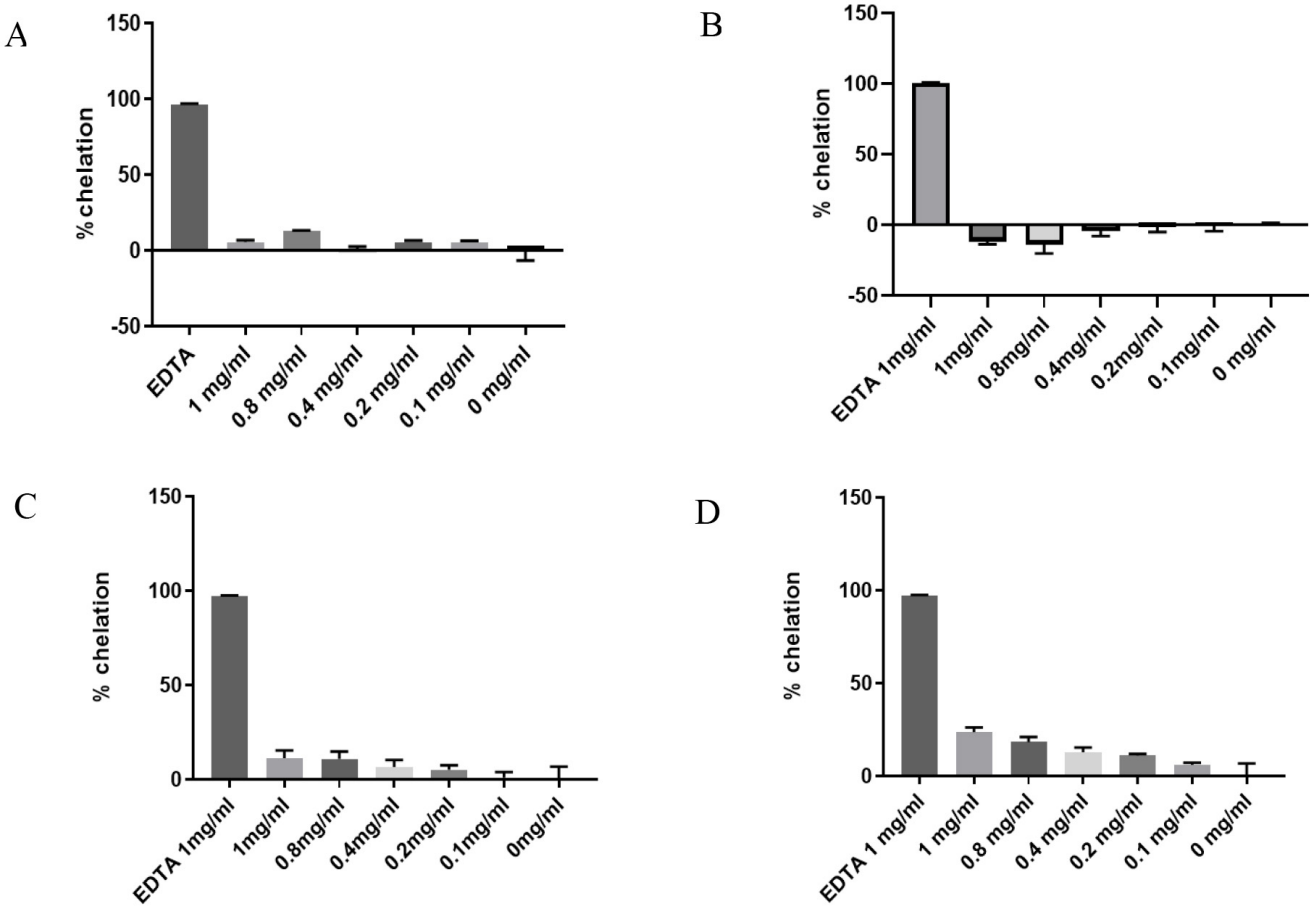
Ferrozine binding assay showing the iron chelation property of the methanolic extracts using EDTA as a reference **(A)** *Carpolobia lutea* bark **(B)** *Carpolobia lutea* leaves **(C)** *Phyllanthus muellerianus* leaves **(D)** *Spondias purpurea* leaves.

Using a ferrozine binding assay, we compared the ability of the selected medicinal plant extracts to chelate iron II relative to Ethylenediaminetetraacetic acid (EDTA) as a standard. Of the three plants tested, *Spondias purpurea* leaf extract (SP) had the highest chelation efficacy with 23% chelation property at 1 mg/ml and 11% at 0.2 mg/ml, respectively. In contrast, *Carpolobia lutea* bark and C. lutea leaf extract had the least chelation efficacy with the leaf extract having negative values. The results are presented in Figure 1. Since SP exhibited moderate iron chelating activity at 0.2 mg/ml, we continued with this concentration to evaluate its therapeutic potential in a cell culture model of PD. SP was also analyzed using liquid chromatography-mass spectrometry, which revealed the detection of flavonoids assigned as tetrahydroxy- and pentahydroxy-flavones, and as gallic and ellagic acid derivatives (Supplementary Table S1).

### 2.2 *In vitro* studies in CAD cells

Cath-a differentiated (CAD) cells are a variant of a catecholaminergic cell line derived from the brain of mice. They express neuron specific proteins and show biochemical and morphological features of primary neurons. They have active tyrosine hydroxylase and accumulate 3,4 Dihydroxyphenylalanine (DOPA)—as such, they have found use as a cell culture model in PD research.

#### 2.2.1 SP protects against 6-hydroxydopamine (6-OHDA) toxicity in CAD cells

**Figure 2:**
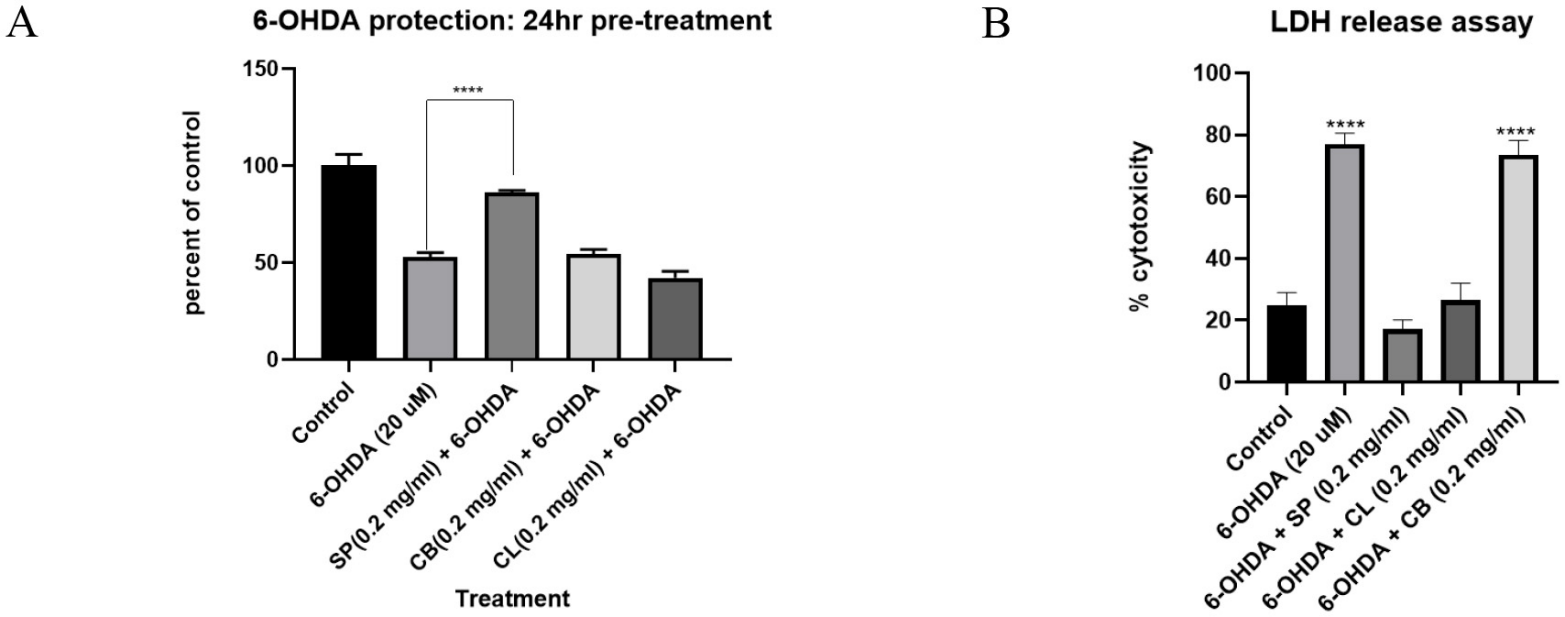
The effect of plant extracts; *Spondias purpurea* leaves (SP), *Carpolobia lutea* bark (CB), and *Carpolobia lutea* leaves (CL) on 6-OHDA induced toxicity in CAD cells. **(A)** MTT assay **(B)** LDH release assay. Data presented as mean ± SEM. Group means were compared using one-way ANOVA and Tukey's *post hoc* test. ****P < 0.0001.

The neurotoxin, 6-hydroxydopamine has a predilection for catecholaminergic neurons and it is commonly used in experimental animal models of PD. Pre-treating CAD cells with SP (0.2 mg/ml) for 24 h before 6-OHDA exposure significantly preserved cell viability as shown by both MTT and lactate dehydrogenase (LDH) release assays (Figure 2).

### 2.3 *In vivo* studies in *Drosophila melanogaster*

#### 2.3.1 SP improves survival of PINK-1 mutant flies

**Figure 3:**
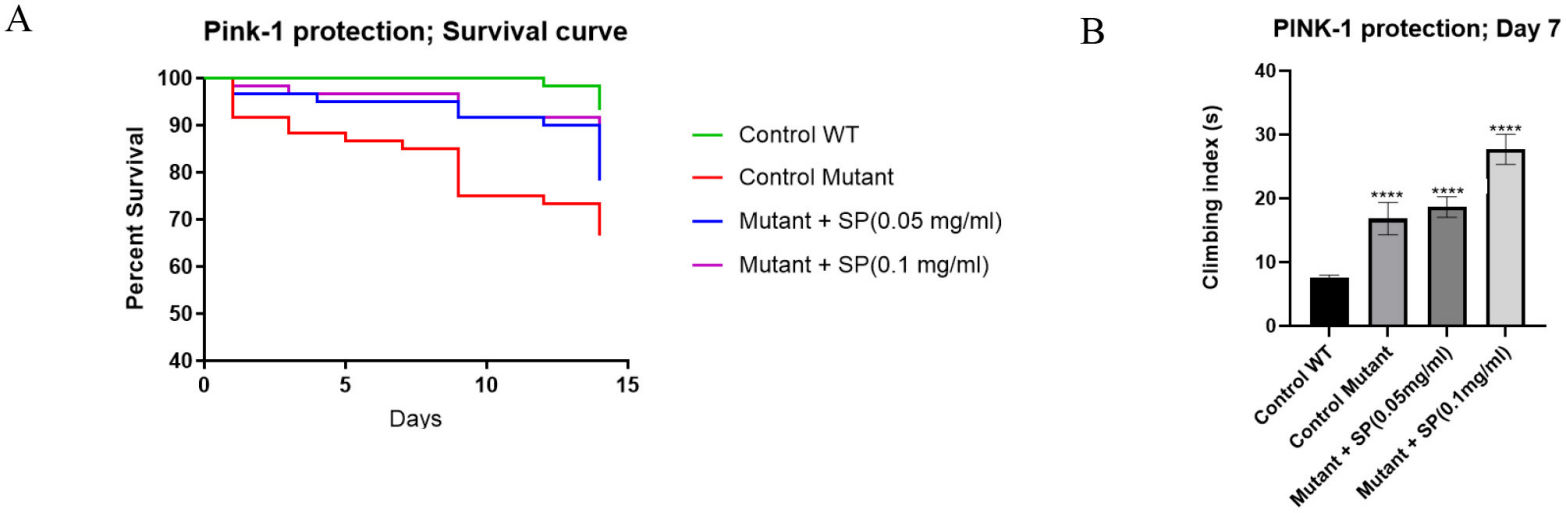
Effect of SP on survival and motor deficits in pink-1 mutant flies (*n* = 60 for all groups): **(A)** survival curve after 2 weeks of treatment. The proportion of control, pink-1, SP (0.05 mg/ml) and SP (0.1 mg/ml) at day 14 was 93.3%, 66.67%, 78.33%, and 81.67%, respectively. The survival curves were compared with a Log-rank (Mantel-Cox) test and found to be significantly different (*P* = 0.0016). Pairwise comparisons with Bonferroni adjusted *p-values* are as follows: Control WT vs. Control mutant, *P* = 0.0012; Control WT vs. Mutant + SP (0.05 mg/ml), *P* = 0.1008; Control WT vs. Mutant + SP (0.1 mg/ml), *P* = 0.2988; **(B)** climbing assay after 7 days of treatment. The pink-1 mutant had significantly worse climbing index compared to the control (*P* < 0.0001) but the SP 0.1 mg/ml supplementation of the feed worsened the performance in the climbing assay while the 0.05 mg/ml had no significant effect. Differences between group means were compared using one way ANOVA and Tukey *post hoc* test. ****P < 0.0001

PTEN-Induced kinase 1 (PINK-1) is an important protein in mitochondrial homeostasis and mutation of its gene is associated with autosomal recessive juvenile Parkinsonism. Pink-1 mutant flies have significant mitochondrial defects particularly in their indirect flight muscles which confers obvious locomotor deficits and reduced survival on the flies. SP supplementation improved survival after two weeks (Figure 3A) but did not ameliorate locomotor deficits as shown in the climbing assay (Figure 3B).

#### 2.3.2 Continuous administration of SP does not improve survival in vanadium-exposed flies

**Figure 4:**
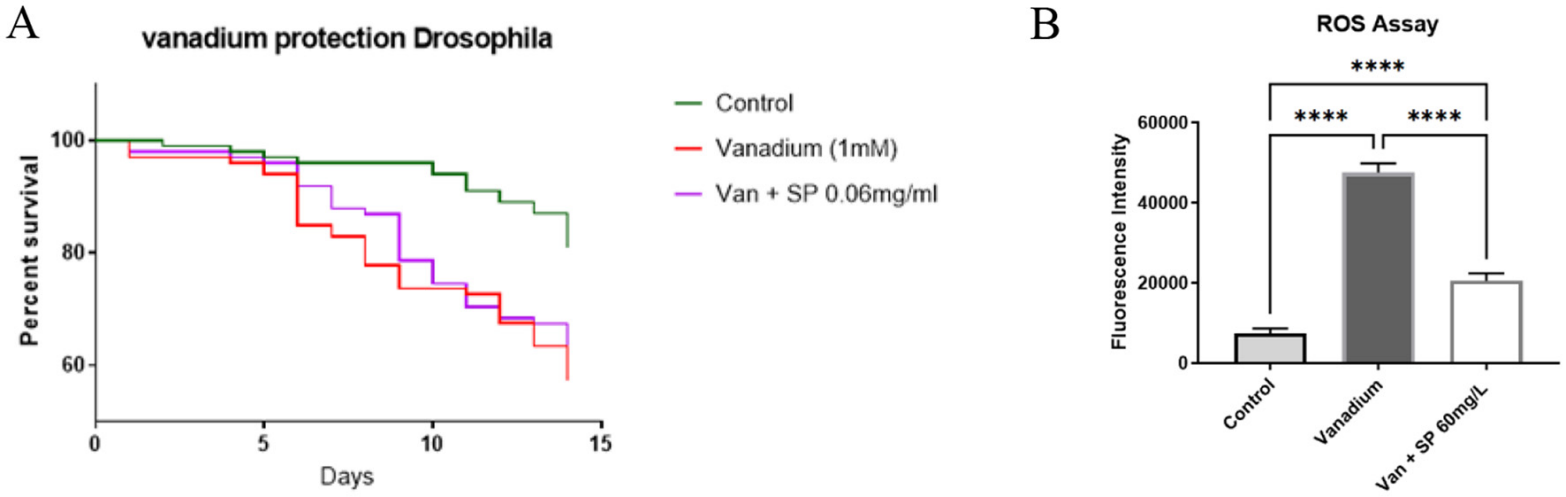
Effect of SP on the survival and generation of reactive oxygen species in vanadium treated flies (*n* = 100 for all groups): **(A)** survival curve after 14 days of treatment. On day 14, the survival proportion of control, vanadium and vanadium + SP (0.06 mg/ml) treated groups were 81%, 57%, and 62%, respectively. The survival curves were compared with Log-rank (Mantel-Cox) test and vanadium treatment significantly reduced survival of the vanadium treated flies (*P* = 0.0007) but SP treatment was unable to mitigate this effect. Pairwise comparisons with Bonferroni adjusted *p-values* are as follows: Control vs. Vanadium (1 mM), *P* = 0.0003; Control vs. Van + SP (0.06 mg/ml), *P* = 0.0057; **(B)** DCFDA assay. After 14 days of exposure, vanadium significantly increased the generation of ROS compared with the control group as shown in this figure. SP treatment significantly reduced vanadium induced ROS generation, although the ROS level did not get as low as that of the control group. Data expressed as mean ± SEM and differences between group means compared using one-way ANOVA and Tukey's *post hoc* test. ****P < 0.0001.

In w1118 flies exposed to 1 mM sodium metavanadate, we observed significant drop in survival in the first 2 weeks of exposure as shown in Figure 4A. From the results shown in Figure 3, since 0.1 mg/ml SP worsened the climbing index but improved the survival of Pink-1 mutant flies, we wanted to see if a slight increase in the dose (from 0.05 to 0.06 mg/ml) could preserve the effect on survival without worsening the climbing index in vanadium exposed flies. Continuous treatment with SP (0.06 mg/ml) appeared to improve survival between day 5 and day 10 but this protection was lost afterwards as survival dropped to the level seen in those exposed to vanadium alone. This was despite the fact that SP treatment reduced the vanadium induced reactive oxygen species generation as shown by the DCFDA assay (Figure 4B). This prompted a change in treatment paradigm where we exposed the vanadium treated flies to intermittent treatment with SP. We speculated that the extract may have accumulated in the flies and exacerbated the toxicity of vanadium.

#### 2.3.3 Intermittent SP treatment improves survival in vanadium exposed flies

**Figure 5:**
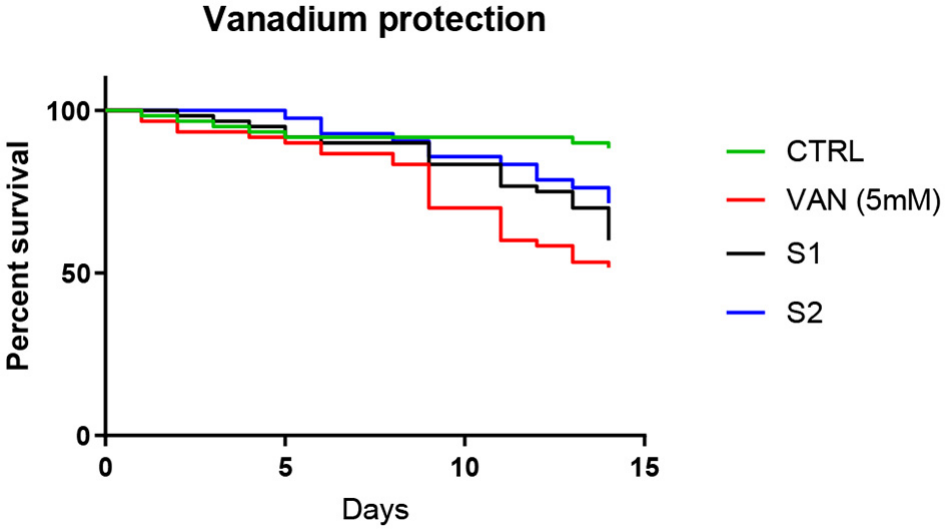
Effect of intermittent SP treatment on survival of vanadium treated flies. Control group (*n* = 60) were raised on instant drosophila feed from day 1 – 15; Vanadium group (*n* = 60) received 5 mM sodium metavanadate from day 1 to day 15; S1 (*n* = 60) received 5 mM sodium metavanadate from day 1–15 as well as 0.05 mg/ml of *Spondias purpurea* extract from day 1–7; S2 (*n* = 42) received 5 mM sodium metavanadate from day 1–15 as well as 0.05 mg/ml of *Spondias purpurea* extract staggered (3 days on, 3 days off) from day 1–15. Survival proportions at day 15 are as follows: control, 88.3%; Vanadium, 51.67%; S1, 60%; S2, 71.43%. The survival curves were compared with Log-rank (Mantel-Cox) test and found to be significantly different (*P* < 0.0001). Pairwise comparisons with Bonferroni adjusted *p-values* are as follows: CTRL vs. VAN, *P* = 0.0006; CTRL vs. S1, *P* = 0.0042; CTRL vs. S2, *P* = 0.246.

**Figure 6:**
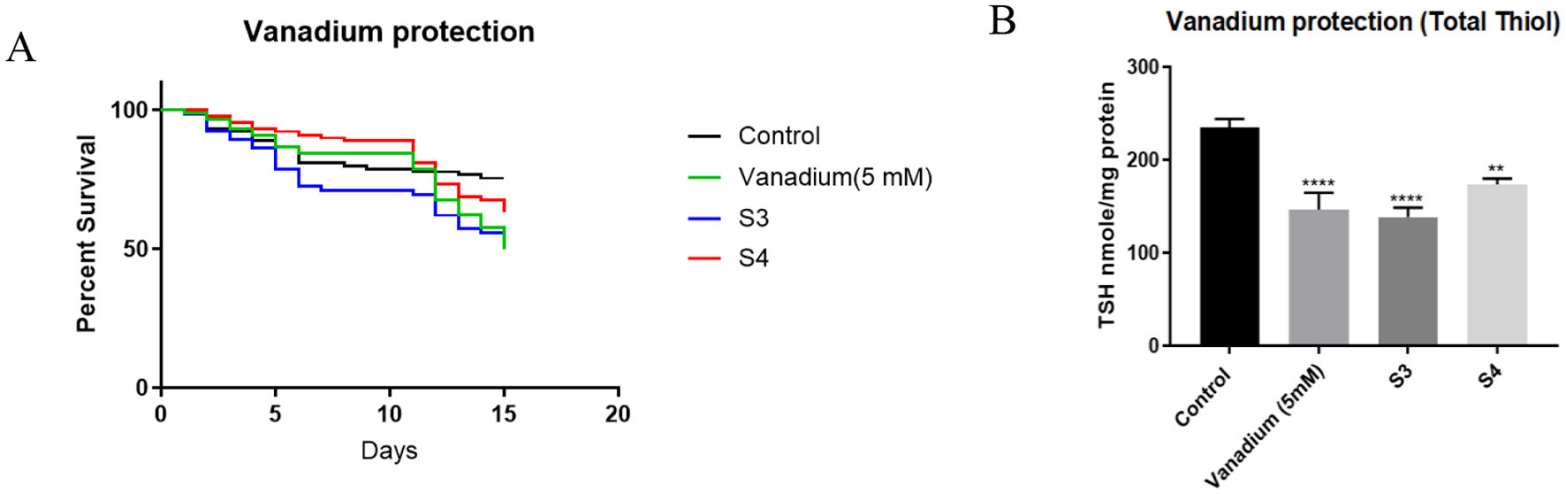
Effect of intermittent SP treatment on survival and total thiol levels of vanadium treated flies **(A)** survival curve. The control group (*n* = 90) were raised on instant drosophila feed from day 1–15; vanadium group (*n* = 90) received 5 mM sodium metavanadate from day 1–15; S3 (*n* = 66) received 5 mM sodium metavanadate from day 1–15 as well as 0.05 mg/ml of *Spondias purpurea* extract from day 7–15; S4 (*n* = 90) received 5 mM sodium metavanadate from day 1–15 as well as 0.05 mg/ml of *Spondias purpurea* extract staggered (3 days on, 3 days off) from day 7–15. Survival proportions at day 15 are as follows: control, 75.5%; Vanadium, 50%; S3, 51.25%; S4, 63.3%. The survival curves were compared with Log-rank (Mantel-Cox) test and found to be significantly different (*P* = 0.0059). Pairwise comparisons with Bonferroni adjusted *p-values* are as follows: Control vs Vanadium (5 mM), *P* = 0.0144; Control vs. S3, *P* = 0.0204; Control vs. S4, *P* = 0.9906. **(B)** Total thiol assay. Data expressed as mean ± SEM. Group means were compared using one-way ANOVA and Tukey's multiple comparisons test. Vanadium caused a statistically significant reduction in the total thiol levels compared to the control group (****P < 0.0001). This reduction was not mitigated by SP although the S4 group showed a positive trend toward recovery (**P = 0.001).

In wildtype fruit flies, a significant improvement in survival following vanadium exposure was observed when SP supplementation of the feed at 0.05 mg/ml was not continuous (Figure 5). This was achieved in two ways: flies in the first intermittent group (S1) received SP supplementation of their diet continuously for the first seven days of vanadium treatment and the SP was withdrawn from day 8–15; the second group of flies, (S2) received SP supplementation of their diet every 3 days (3 days on, 3 days off). When intermittent SP treatment of vanadium exposed flies was restricted to only the second week, the effect on survival was not as significant as when the intermittent treatment started from the first week (Figure 6), and the antioxidant thiol level showed a trend toward improvement relative to the vanadium only group.

## 3 Discussion

Iron plays a role in the major cellular pathologies associated with PD (26) and the ability to chelate iron is useful to select plant species for further evaluation as a potential therapeutic agent in PD. The methanolic extract of *Carpolobia lutea* G. Don leaves had negative chelation results in the ferrozine binding assay, which suggests the presence of iron in the leaves. Indeed, Ayoola et al. (37) reported an iron concentration of 449.54 ± 0.02 ppm in *C*. *lutea* leaves. It can therefore be speculated that *C*. *lutea* leaves could potentially exacerbate PD symptoms if administered to PD patients. Iron has numerous important physiological roles in the body. It is important in mitochondrial respiration, myelin synthesis, oxygen transport, and neurotransmitter synthesis, for example (14). A strong iron chelator could therefore be counterproductive in the management of PD. The mild chelating activity of SP at 0.2 mg/ml is therefore desirable, as it may mitigate pathological iron accumulation without disrupting essential physiological functions.

6-Hydroxydopamine is a neurotoxin selective for catecholaminergic neurons, and its specificity makes it useful in experimental models of PD (38). CAD cells, being catecholaminergic cells are sensitive to 6-OHDA. The mechanism of toxicity is complex and uncertain. Putative mechanisms include: inhibition of mitochondrial function, generalized protein modifications leading to endoplasmic reticulum stress, release of Fe(II) from intracellular stores, and generation of reactive oxygen species (39). The ability of SP to chelate iron is likely behind the protection from 6-OHDA toxicity in CAD cells that was observed. Other iron chelators have also been effective in ameliorating 6-OHDA-induced neurotoxicity (40).

Pink-1 mutation is associated with autosomal recessive juvenile Parkinsonism, a disease characterized by motor deficits and dopaminergic neurodegeneration. Studies in Pink-1 mutant *Drosophila* have shown that mitochondrial dysfunction underlies the major degenerative changes observed in those flies. We observed that SP ameliorated the impaired survival, but not the long-term locomotor deficits in Pink-1 mutant flies. Perhaps the two phenotypes are influenced by different pathways or the motor deficits are already established and only short-term benefits can be seen. The locomotor deficits observed in Pink-1 mutant flies primarily result from mitochondrial dysfunction in the muscles, as reported by (41). They documented that locomotor deficits can be seen as early as 3 days in Pink-1 mutant flies, whereas dopaminergic neuronal loss is not observed until the flies reach 30 days of age. Liu et al. (42) reported amelioration of both survival and motor deficits when treatment started from larval stage of development which suggests that structural defects are perhaps more difficult to remedy completely once formed. The mitochondrial dysfunction in Pink-1 flies makes them prone to oxidative stress and this is supported by the lower TSH levels relative to control. SP treatment showed a trend toward improvement in TSH levels.

The role of reactive oxygen species in vanadium toxicity is well established (10, 43, 44), and the ability of vanadium to induce ROS is linked to its ability to disrupt iron homeostasis. Vanadium is reported to release iron from ferritin, and desferroxamine, an iron chelator, has been shown to protect against vanadium induced neurotoxicity (32). The iron chelating ability of SP is probably responsible for its ability to improve the survival of vanadium exposed *Drosophila melanogaster*. This property was observed for other plant products, such as *Aloysia citrodora* Paláu, with modest iron chelating properties (45). This protection is only evident when the dosing is intermittent rather than continuous. We hypothesize that the extracts may be accumulating in the body. This is suggested by the observation that the S2 and S4 groups, which received the extract for 3 days followed by 3 days without it (alternating), had better outcomes compared to the S1 and S3 groups, which received the extract continuously for seven days. These findings suggest that continuous administration of SP may exacerbate vanadium toxicity, and requires further investigation.

## 4 Materials and methods

### 4.1 Plant extracts

Methanolic extracts of the leaves of *Carpolobia lutea* G. Don, *Spondias purpurea* L., *Phyllanthus muellerianus* (Kuntze) Exell and the bark of *C. lutea* were kind donations from Prof Elufioye of the Department of Pharmacognosy, University of Ibadan.

The provenance details are as follows:

*Spondias purpurea* L.

The leaves were collected from Oworo District of Lokoja Local Government Area, Kogi State, Nigeria in April 2016. The plant was identified by Mr. A. Adeyemo of Forest Herbarium Ibadan (FHI), where a voucher specimen (FHI 110431) was deposited.

*Phyllanthus muellerianus* (Kuntze) Exell

The leaves were collected in Enugu State, Nigeria in November 2016, and authenticated at Forestry Herbarium Ibadan (FHI), where a voucher specimen, with voucher number FHI 111339 was deposited.

*Carpolobia lutea* G. Don

The leaves of *Carpolobia lutea* were collected from Aba Oluwo Forest in Ibadan, Nigeria in February 2020 and authenticated at Forestry Herbarium Ibadan (FHI), where a voucher specimen, with voucher number FHI 112847 was deposited.

The bioactive *S. purpurea* methanolic extract was analyzed using liquid chromatography-mass spectrometry (the LC-MS method is described in the Supplementary material). Before use in cell culture studies, the extracts were dissolved in 2.5% DMSO and sterilized by filtration through a 0.2 μm Millipore filter.

### 4.2 Ferrozine binding assay

To assess the iron chelating property of the extracts, a ferrozine binding assay was carried out using a method adapted from (46). Briefly, different concentrations of the extracts were prepared in methanol (0, 0.1, 0.2, 0.4, 0.8, and 1 mg/ml). To 1 ml of each solution, 50 μl of 2 mM FeCl_2_ was added. One mg/ml sodium EDTA was used as a positive control (standard). Subsequently, 200 μl of 5 mM ferrozine was added and the mixture was vortexed and left to sit at room temperature on the bench for 10 min. Two hundred μl aliquots of the solution were transferred in triplicate to 96 well plates and the absorbance was read at 562 nm on a spectrophotometer. The percentage inhibition of Fe^2+^-ferrozine complex formation was calculated as: $\left(\frac{Ac-As}{Ac}\right)\times \;100$.

Where Ac is absorbance of control and As is the absorbance of the sample or standard.

### 4.3 CAD cell culture

CAD cells were grown as described by (47); the cells were grown in Dulbecco modified eagle's medium (DMEM/F-12) supplemented with 10% fetal bovine serum (FBS) and kept in an incubator set at 37 °C and 5% CO2 passaging was done at 1:5 dilution every 4–5 days.

### 4.4 CAD cell treatment with 6-hydroxydopamine and plant extracts

Cells are dislodged from flasks at 70%−80% confluency by gentle pipetting. The cell suspension was transferred into 15 ml tubes and centrifuged at 200 g at 4 °C for 5 min. the pellet was resuspended at 1:4 dilution and the cells were plated in 24-well plates and grown for 24 h in the incubator. After 24 h, the media was carefully removed and replaced with fresh media containing the different treatment substances as described below:

a) Determination of doses: To determine which dose of the extracts to use, the cells were exposed to different concentrations (0.2, 0.4, 0.6, 0.8, and 1 mg/ml). The LD_50_ of 6-OHDA was determined by exposing the cells to 10 μM, 20 μM, 40 μM, 60 μM, and 80 μM of 6-OHDA. After 24 h, MTT assays were carried out to determine viability of the cells. The results are presented in Supplementary Figures S1, S2.
b) Neuroprotection study: Cells were pretreated with the chosen extract concentration for 24 h followed by introduction of 20 μM 6-OHDA. After an additional 24 h, MTT and LDH assays were carried out to assess the protective effects of the plant extracts against 6-OHDA toxicity.

### 4.5 MTT assay

Cell viability was tested using the MTT assay as described (47). Briefly, 5 mg/ml MTT solution in phosphate buffered saline was added to the cells. The cells were then returned into the incubator for 150 min. Subsequently, the MTT-containing medium was removed and the cells were rinsed with PBS, and isopropanol was added to dissolve the formazan product. Sample absorbance was measured at 595 nm on a spectrophotometer.

### 4.6 LDH release assay

The CytoTox 96 non-radioactive assay kit from Promega was used to quantify lactate dehydrogenase (LDH) released from the cells. This was done according to the manufacturer's instruction.

### 4.7 Fly stocks and culture conditions

*Drosophila melanogaster* strains used in this study included Harwich, Dahomey, w^1118^ wild-type and PINK-1 mutant (w-PINK-1 B9/FM7.GFPw+). The flies were kept in an incubator at 25 °C and 12-h light: dark cycle. Flies were fed instant *Drosophila* medium (Formula 4–24^®^, Carolina Biological Supply Company, Burlington NC, USA) according to the manufacturer's instructions. Flies were transferred into new treatment vials with fresh feed every 3 days.

### 4.8 *Drosophila* treatment

Male flies aged 1–3 days were selected under CO_2_ anesthesia into treatment vials containing fresh feed mixed with the appropriate treatment substances. Each treatment group contained 5 vials with 20 flies per vial. Each experiment was for 14 days except otherwise stated. Flies were transferred into new treatment vials every 3 days. Dead flies were counted daily to compute survival curves. On day 7, a climbing assay was carried out as described by (41).

### 4.9 Biochemical assays on fly heads

At the end of the experiments, flies were transferred into an empty 15 ml centrifuge tube and snap-frozen by immersion liquid nitrogen. The tubes were then vortexed to decapitate the frozen flies. Fly heads were transferred into pre-weighed Eppendorf tubes and homogenized in 0.1 M phosphate buffered saline (PBS) and subsequently centrifuged for 10 min at 4,000 × g at 4 °C. The supernatant was stored at −20 °C for downstream applications:

a. Total thiol measurement: This was carried out using the method described by (48). Twenty μl of the sample, 10 μl of DTNB and 170 μl of 0.1 M PBS constituted the reaction mixture. The mixture was incubated for 30 min on the benchtop after which the sample absorbance was read at 412 nm on a spectrophotometer. Reduced glutathione was used as a standard and the total thiol concentration was expressed as nmol/mg of protein in the sample.
b. Reactive Oxygen Species Assay: ROS levels in fly samples were detected using the dichlorofluorescein diacetate (DCFDA) assay as described by (45). The fluorescent product of DCFDA oxidation was measured at 30 s interval for 10 min on a fluorescent spectrophotometer (BioTek synergy H1 multimode reader). Excitation/emission wavelengths were set at 488 nm/525 nm. The rate of DCF formation was expressed as a percentage of the control group.

## Data Availability

The original contributions presented in the study are included in the article/Supplementary material, further inquiries can be directed to the corresponding authors.

## References

[1] United Nations. World population ageing, 2014. Dep Econ Soc Aff Popul Div. (2014) 73.

[2] Musillo C Borgi M Saul N Möller S Luyten W Berry A. Natural products improve healthspan in aged mice and rats: a systematic review and meta-analysis. Neurosci Biobehav Rev. (2021) 121:89–105. 10.1016/j.neubiorev.2020.12.001 33309907

[3] Hou Y Dan X Babbar M Wei Y Hasselbalch SG Croteau DL. Ageing as a risk factor for neurodegenerative disease. Nat Rev Neurol. (2019) 15:565–81. 10.1038/s41582-019-0244-7 31501588

[4] Vermunt L Sikkes SAM Hout A Handels R Bos I Flier WM. Duration of preclinical, prodromal, and dementia stages of Alzheimer's disease in relation to age, sex, and APOE genotype. Alzheimer's Dement. (2019) 15:888–98. 10.1016/j.jalz.2019.04.001 31164314 PMC6646097

[5] Calderón-Garcidueñas L Avila-Ramírez J Calderón-Garcidueñas A González-Heredia T Acuña-Ayala H Chao CK. Cerebrospinal fluid biomarkers in highly exposed PM2.5 urbanites: the risk of Alzheimer's and Parkinson's diseases in young Mexico city residents. Lewczuk P, editor. J Alzheimer's Dis. (2016) 54:597–613. 10.3233/JAD-160472 27567860

[6] Calderón-Garcidueñas L Serrano-Sierra A Torres-Jardón R Zhu H Yuan Y Smith D. The impact of environmental metals in young urbanites' brains. Exp Toxicol Pathol. (2013) 65:503–11. 10.1016/j.etp.2012.02.006 22436577 PMC3383886

[7] Afeseh Ngwa H Kanthasamy A Anantharam V Song C Witte T Houk R. Vanadium induces dopaminergic neurotoxicity via protein kinase Cdelta dependent oxidative signaling mechanisms: relevance to etiopathogenesis of Parkinson's disease. Toxicol Appl Pharmacol. (2009) 240:273–85. 10.1016/j.taap.2009.07.025 19646462 PMC2753722

[8] Zwolak I. Protective effects of dietary antioxidants against vanadium-induced toxicity: a review. Oxid Med Cell Longev. (2020) 2020:1–14. 10.1155/2020/1490316 31998432 PMC6973198

[9] Li C Wu C Zhang J Li Y Zhang B Zhou A. Associations of prenatal exposure to vanadium with early-childhood growth: a prospective prenatal cohort study. J Hazard Mater. (2021) 411:125102. 10.1016/j.jhazmat.2021.125102 33461012

[10] Fatola OI Olaolorun FA Olopade FE Olopade JO. Trends in vanadium neurotoxicity. Brain Res Bull. (2019) 145:75–80. 10.1016/j.brainresbull.2018.03.010 29577939

[11] Folarin OR Snyder AM Peters DG Olopade F Connor JR Olopade JO. Brain metal distribution and neuro-inflammatory profiles after chronic vanadium administration and withdrawal in mice. Front Neuroanat. (2017) 11:1–15. 10.3389/fnana.2017.00058 28790895 PMC5524677

[12] Jhang KA Park JS Kim HS Chong YH. Resveratrol ameliorates tau hyperphosphorylation at Ser396 site and oxidative damage in rat hippocampal slices exposed to vanadate: implication of ERK1/2 and GSK-3β signaling cascades. J Agric Food Chem. (2017) 65:9626–34. 10.1021/acs.jafc.7b03252 29022339

[13] Dorsey ER Sherer T Okun MS Bloem BR. The emerging evidence of the Parkinson pandemic. J Parkinsons Dis. (2018) 8:S3–8. 10.3233/JPD-181474 30584159 PMC6311367

[14] Belaidi AA Bush AI. Iron neurochemistry in Alzheimer's disease and Parkinson's disease: targets for therapeutics. J Neurochem. (2016) 139:179–97. 10.1111/jnc.13425 26545340

[15] Lotharius J Brundin P. Pathogenesis of parkinson's disease: dopamine, vesicles and α-synuclein. Nat Rev Neurosci. (2002) 3:932–42. 10.1038/nrn983 12461550

[16] Nunez M Chana-Cuevas P. New perspectives in iron chelation therapy for the treatment of Parkinson's disease. Neural Regen Res. (2019) 14:1905. 10.4103/1673-5374.259614 31290444 PMC6676885

[17] Kulisevsky J Oliveira L Fox SH. Update in therapeutic strategies for Parkinson's disease. Curr Opin Neurol. (2018) 31:439–47. 10.1097/WCO.0000000000000579 29746402

[18] Hare DJ Arora M Jenkins NL Finkelstein DI Doble PA Bush AI. Is early-life iron exposure critical in neurodegeneration? Nat Rev Neurol. (2015) 11:536–44. 10.1038/nrneurol.2015.100 26100754

[19] Ward RJ Zucca FA Duyn JH Crichton RR Zecca L. The role of iron in brain ageing and neurodegenerative disorders. Lancet Neurol. (2014) 13:1045–60. 10.1016/S1474-4422(14)70117-6 25231526 PMC5672917

[20] Barnham KJ Masters CL Bush AI. Neurodegenerative diseases and oxidative stress. Nat Rev Drug Discov. (2004) 3:205–14. 10.1038/nrd1330 15031734

[21] Pyatigorskaya N Sharman M Corvol JC Valabregue R Yahia-Cherif L Poupon F. High nigral iron deposition in LRRK2 and Parkin mutation carriers using R2^*^ relaxometry. Mov Disord. (2015) 30:1077–84. 10.1002/mds.26218 26011561

[22] Hirsch EC Brandel J-P Galle P Javoy-Agid F Agid Y. Iron and aluminum increase in the substantia nigra of patients with Parkinson's Disease: an X-ray microanalysis. J Neurochem. (1991) 56:446–51. 10.1111/j.1471-4159.1991.tb08170.x 1988548

[23] Uversky VN Li J Fink AL. Metal-triggered structural transformations, aggregation, and fibrillation of human α-synuclein. J Biol Chem. (2001) 276:44284–96. 10.1074/jbc.M105343200 11553618

[24] Xiao Y Chen X Huang S Li G Mo M Zhang L. Iron promotes α-synuclein aggregation and transmission by inhibiting TFEB-mediated autophagosome-lysosome fusion. J Neurochem. (2018) 145:34–50. 10.1111/jnc.14312 29364516

[25] Bartels M Weckbecker D Kuhn PH Ryazanov S Leonov A Griesinger C. Iron-mediated aggregation and toxicity in a novel neuronal cell culture model with inducible alpha-synuclein expression. Sci Rep. (2019) 9:9100. 10.1038/s41598-019-45298-6 31235814 PMC6591385

[26] Kim Y Connor JR. The roles of iron and HFE genotype in neurological diseases. Mol Aspects Med. (2020) 75:100867. 10.1016/j.mam.2020.100867 32654761

[27] Connor JR Snyder BS Arosio P Loeffler DA LeWitt P. A quantitative analysis of isoferritins in select regions of aged, Parkinsonian, and Alzheimer's diseased brains. J Neurochem. (2002) 65:717–24. 10.1046/j.1471-4159.1995.65020717.x 7616228

[28] Howitt J Gysbers AM Ayton S Carew-Jones F Putz U Finkelstein DI. Increased Ndfip1 in the substantia nigra of parkinsonian brains is associated with elevated iron levels. PLoS ONE. (2014) 24:e87119. 10.1371/journal.pone.0087119 24475238 PMC3901732

[29] Park J Lee DG Kim B Park SJ Kim JH Lee SR. Iron overload triggers mitochondrial fragmentation via calcineurin-sensitive signals in HT-22 hippocampal neuron cells. Toxicology. (2015) 337:39–46. 10.1016/j.tox.2015.08.009 26318285

[30] Urrutia PJ Aguirre P Tapia V Carrasco CM Mena NP Núñez MT. Cell death induced by mitochondrial complex I inhibition is mediated by iron regulatory protein 1. Biochim Biophys Acta Mol Basis Dis. (2017) 1863:2202–9. 10.1016/j.bbadis.2017.05.015 28502703

[31] Ward RJ Dexter DT Martin-Bastida A Crichton RR. Is chelation therapy a potential treatment for Parkinson's Disease? Int J Mol Sci. (2021) 22:3338. 10.3390/ijms22073338 33805195 PMC8036775

[32] Todorich B Olopade JO Surguladze N Zhang X Neely E Connor JR. The mechanism of vanadium-mediated developmental hypomyelination is related to destruction of oligodendrocyte progenitors through a relationship with ferritin and iron. Neurotox Res. (2011) 19:361–73. 10.1007/s12640-010-9167-1 20237879

[33] Usende IL Leitner DF Neely E Connor JR Olopade JO. The deterioration seen in myelin related morphophysiology in vanadium exposed rats is partially protected by concurrent iron deficiency. Niger J Physiol Sci. (2016) 31:11–22. 27574759

[34] Nwidu LL Elmorsy E Thornton J Wijamunige B Wijesekara A Tarbox R. Anti-acetylcholinesterase activity and antioxidant properties of extracts and fractions of Carpolobia lutea. Pharm Biol. (2017) 55:1875–83. 10.1080/13880209.2017.1339283 28629287 PMC6130458

[35] Onoja J Elufioye T. BRITISH JOURNAL OF PHARMACEUTICAL AND MEDICAL RESEARCH anti-Alzheimer's potential of stigmasterol isolated from phyllanthus muellerianus (Kuntze) exell and molecular docking study. (2023) 3921–36. 10.24942/bjpmr.2022.993

[36] Elufioye TO Berida TI GC-MS. Analysis and antioxidant activity of spondias purpurea L (*Anacardiaceae*). Pharmacogn J. (2018) 10:941–5. 10.5530/pj.2018.5.159

[37] Ayoola GA Shonekan OO Johnson OO Adeoti B. Determination of the selected heavy metals in carpolobia lutea, G. Don *(Polygalaceae)* leaves and fruits. J Chem Soc Niger. (2018) 43:31–5.

[38] Glinka Y Gassen M Youdim MBH. Mechanism of 6-hydroxydopamine neurotoxicity. J Neural Transm Suppl. (1997) 50:55–66. 10.1007/978-3-7091-6842-4_7 9120425

[39] Varešlija D Tipton KF Davey GP McDonald AG. 6-Hydroxydopamine: a far from simple neurotoxin. J Neural Trans. (2020) 127:213–30. 10.1007/s00702-019-02133-6 31894418

[40] Youdim MBH Stephenson G Shachar DB. Ironing iron out in Parkinson's disease and other neurodegenerative diseases with iron chelators: a lesson from 6-hydroxydopamine and iron chelators, desferal and VK-28. Ann NY Acad Sci. (2004) 1012:306–25. 10.1196/annals.1306.025 15105275

[41] Park J Lee SB Lee S Kim Y Song S Kim S. Mitochondrial dysfunction in Drosophila PINK1 mutants is complemented by parkin. Nature. (2006) 441:1157–61. 10.1038/nature04788 16672980

[42] Liu M Yu S Wang J Qiao J Liu Y Wang S. Ginseng protein protects against mitochondrial dysfunction and neurodegeneration by inducing mitochondrial unfolded protein response in Drosophila melanogaster PINK1 model of Parkinson's disease. J Ethnopharmacol. (2020) 247:112213. 10.1016/j.jep.2019.112213 31562951

[43] Valko M Morris H Cronin M. Metals, toxicity and oxidative stress. Curr Med Chem. (2005) 12:1161–208. 10.2174/0929867053764635 15892631

[44] Scibior A Pietrzyk Ł Plewa Z Skiba A. Vanadium: Risks and possible benefits in the light of a comprehensive overview of its pharmacotoxicological mechanisms and multi-applications with a summary of further research trends. J Trace Elem Med Biol. (2020) 61:126508. 10.1016/j.jtemb.2020.126508 32305626 PMC7152879

[45] Ohiomokhare S Olaolorun F Ladagu A Olopade F Howes MJR Okello E. The pathopharmacological interplay between vanadium and iron in Parkinson's disease models. Int J Mol Sci. (2020) 21:6719. 10.3390/ijms21186719 32937783 PMC7554808

[46] Liu J Wang C Wang Z Zhang C Lu S Liu J. The antioxidant and free-radical scavenging activities of extract and fractions from corn silk (*Zea mays* L.) and related flavone glycosides. Food Chem. (2011) 126:261–9. 10.1016/j.foodchem.2010.11.014

[47] Abuhamdah S Abuhamdah R Howes MJR Al-Olimat S Ennaceur A Chazot PL. Pharmacological and neuroprotective profile of an essential oil derived from leaves of Aloysia citrodora Palau. J Pharm Pharmacol. (2015) 67:1306–15. 10.1111/jphp.12424 25877296

[48] Ibraheem O Oyewole TA Adedara A Abolaji AO Ogundipe OM Akinyelu J.. Ackee (Blighia *sapida* K.D. Koenig) leaves and arils methanolic extracts ameliorate CdCl2-induced oxidative stress biomarkers in *Drosophila melanogaster*. Oxid Med Cell Longev. (2022) 2022:1–20. 10.1155/2022/3235031 36425055 PMC9679428

