# Supplementary material for: Iron chelation as a therapeutic target in vanadium neurotoxicity and Parkinson's disease: role of medicinal plants
Source: Front Neurol. 2025 Oct 24;16:1667943. doi: 10.3389/fneur.2025.1667943 (PMC12591949; doi:10.3389/fneur.2025.1667943)
Supplement: Supplementary file 1 [file Data_Sheet_1.pdf]

Supplementary Material

1.1 Supplementary Figures

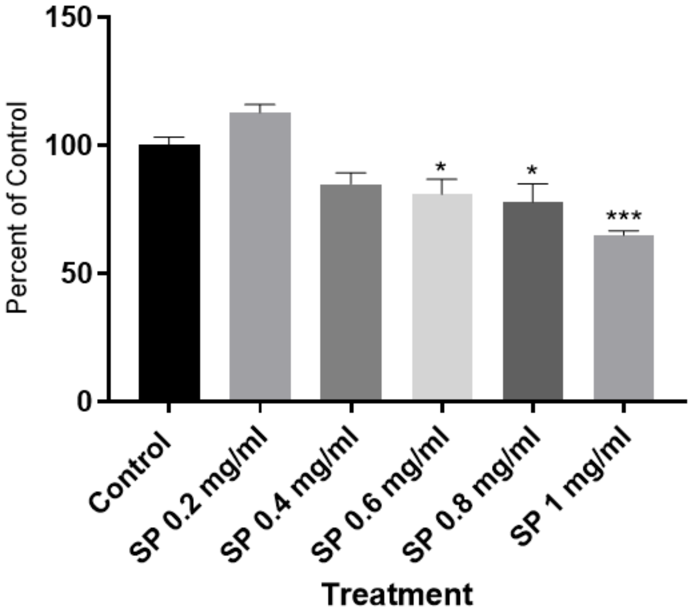

Supplementary Figure 1. Dose response of *Spondias purpurea* leaf extract in undifferentiated CAD cells. Data presented as mean ±SEM. Group means were compared with one-way ANOVA with Dunnett's multiple comparison to compare the group means relative to the control group. *** $P = 0.0002$; * $P < 0.05$

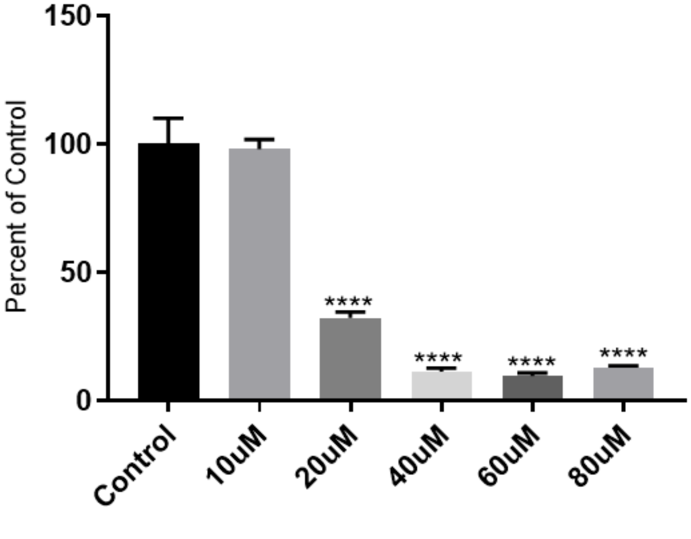

Supplementary Figure 2. Dose response of 6-Hydroxydopamine in undifferentiated CAD cells. Data presented as mean ±SEM. Group means were compared with one-way ANOVA with Dunnett's multiple comparison to compare the group means relative to the control group. **** $P < 0.0001$
